# Imaging Metformin Efficacy as Add-On Therapy in Cells and Mouse Models of Human EGFR Glioblastoma

**DOI:** 10.3389/fonc.2021.664149

**Published:** 2021-05-03

**Authors:** Silvia Valtorta, Alessia Lo Dico, Isabella Raccagni, Cristina Martelli, Valentina Pieri, Paolo Rainone, Sergio Todde, Bastian Zinnhardt, Elisabetta De Bernardi, Angela Coliva, Letterio S. Politi, Thomas Viel, Andreas H. Jacobs, Rossella Galli, Luisa Ottobrini, Valentina Vaira, Rosa Maria Moresco

**Affiliations:** ^1^ Department of Medicine and Surgery and Tecnomed Foundation, University of Milano – Bicocca, Monza, Italy; ^2^ Institute of Molecular Bioimaging and Physiology, National Research Council (IBFM-CNR), Segrate, Italy; ^3^ Nuclear Medicine Department, IRCCS San Raffaele Scientific Institute, Milan, Italy; ^4^ Department of Pathophysiology and Transplantation (DEPT), University of Milan, Milan, Italy; ^5^ SYSBIO Centre of Systems Biology ISBE.ITALY, University of Milano - Bicocca, Milan, Italy; ^6^ Neural Stem Cell Biology Unit, Division of Neuroscience, IRCCS San Raffaele Scientific Institute, Milan, Italy; ^7^ European Institute for Molecular Imaging (EIMI), University of Münster, Münster, Germany; ^8^ Department of Nuclear Medicine, University Hospital Münster, Münster, Germany; ^9^ Department of Biomedical Sciences, Humanitas University, Rozzano, Italy; ^10^ Department of Neuroradiology, Humanitas Clinical and Research Center IRCCS, Rozzano, Italy; ^11^ PARCC, INSERM, Université de Paris, Paris, France; ^12^ Division of Pathology, Fondazione IRCCS Ca’ Granda Ospedale Maggiore Policlinico, Milan, Italy

**Keywords:** GBM - glioblastoma multiforme, metformin, inflammation, PET imaging, [^18^F]FLT, EGFR - epidermal growth factor receptor, TSPO

## Abstract

Glioblastoma (GBM) is a highly aggressive tumor of the brain. Despite the efforts, response to current therapies is poor and 2-years survival rate ranging from 6-12%. Here, we evaluated the preclinical efficacy of Metformin (MET) as add-on therapy to Temozolomide (TMZ) and the ability of [^18^F]FLT (activity of thymidine kinase 1 related to cell proliferation) and [^18^F]VC701 (translocator protein, TSPO) Positron Emission Tomography (PET) radiotracers to predict tumor response to therapy. Indeed, TSPO is expressed on the outer mitochondrial membrane of activated microglia/macrophages, tumor cells, astrocytes and endothelial cells. TMZ-sensitive (Gli36ΔEGFR-1 and L0627) or -resistant (Gli36ΔEGFR-2) GBM cell lines representative of classical molecular subtype were tested *in vitro* and *in vivo* in orthotopic mouse models. Our results indicate that *in vitro*, MET increased the efficacy of TMZ on TMZ-sensitive and on TMZ-resistant cells by deregulating the balance between pro-survival (*bcl2*) and pro-apoptotic (*bax/bad*) Bcl-family members and promoting early apoptosis in both Gli36ΔEGFR-1 and Gli36ΔEGFR-2 cells. *In vivo*, MET add-on significantly extended the median survival of tumor-bearing mice compared to TMZ-treated ones and reduced the rate of recurrence in the TMZ-sensitive models. PET studies with the cell proliferation radiopharmaceutical [^18^F]FLT performed at early time during treatment were able to distinguish responder from non-responder to TMZ but not to predict the duration of the effect. On the contrary, [^18^F]VC701 uptake was reduced only in mice treated with MET plus TMZ and levels of uptake negatively correlated with animals’ survival. Overall, our data showed that MET addition improved TMZ efficacy in GBM preclinical models representative of classical molecular subtype increasing survival time and reducing tumor relapsing rate. Finally, results from PET imaging suggest that the reduction of cell proliferation represents a common mechanism of TMZ and combined treatment, whereas only the last was able to reduce TSPO. This reduction was associated with the duration of treatment response. TSPO-ligand may be used as a complementary molecular imaging marker to predict tumor microenvironment related treatment effects.

## Introduction

Glioblastoma (GBM) represents the most common and aggressive malignant brain neoplasm in adults with no effective treatments. Surgical resection and concomitant radiotherapy followed by adjuvant Temozolomide (TMZ) (Stupp protocol) represent the gold standard for GBM treatment (1). Nevertheless, resistance to TMZ and/or disease progression invariably occur in GBM independently of O6-methylguanine-DNA methyltransferase (MGMT) presence (2) leading to a poor clinical outcome and a median overall-survival of 14.6 months. For this reason, novel treatment approaches for GBM represent an unmet medical need (3). Despite the efforts, the majority of new therapeutic strategies proposed, including targeted-based therapy, showed limited efficacy in clinical trials. The lack of success of existing or newly developed therapy is based on several factors, including the biological complexity and the clonal heterogeneity of GBM. A common hallmark of GBM is represented by an aberrant metabolic phenotype characterized by increased glucose demand and aerobic glycolysis (the so-called Warburg effect) (4). In addition, many of the oncogenes and tumor suppressor proteins, commonly mutated in GBM, regulate cancer metabolism leading to an increased glucose uptake, the switch to the Warburg effect, *de novo* lipogenesis and other alternative metabolic pathways. For the reason above, targeting tumor metabolism represents an attractive therapeutic strategy for GBM (5, 6) particularly using combined strategies (7). The oral antidiabetic Metformin (MET), that modulates 5’ AMP-activated protein kinase (AMPK) and mitochondrial functions, showed promising *in vitro* and *in vivo* results in different types of cancer, including GBM (8–10). MET was initially proposed as a single regimen against glioma-initiating stem cells, however, we and other groups demonstrated that MET is synergic with TMZ and is able to revert TMZ resistance in some mouse models of GBM (11–13). Another negative hallmark of glioma is represented by the high variability of molecular phenotypes. Using an unsupervised hierarchical clustering analysis, Verhaak et al. classified GBM in four molecular subtypes, named Classical, Mesenchymal, Neural and Proneural (14). The four subtypes differ for rate of progression, response to chemotherapy and for molecular signature. The Epidermal Growth Factor Receptor (EGFR) amplification or mutation is present in approximately 57% of tumors, particularly the classical subtype (15). Approximately 50% of tumors carrying EGFR amplification present a specific highly oncogenic and constitutively activated mutant (EGFRvIII, also known as EGFR type III, de2-7, ΔEGFR) (16). Overall, the hyper-activated EGFR phenotype favors treatment resistance and poor clinical outcome (17).

Despite the major role in cell growth, the clinical efficacy of EGFR tyrosine kinase inhibitors was poor. Interestingly, Ciaglia et al. showed that activation of the metabolic sensor AMPK through the administration of N6‐isopentenyladenosine (iPA) inhibited the *in vivo* growth of GBM tumors, with markedly enhanced efficacy in cells with higher levels of EGFR expression/activity (18). Another important point is that EGFR favors a highly inflammatory microenvironment in GBM (19, 20). Although the role of inflammation in glioma is not completely understood, several studies on immune check-point inhibitors suggest a link between inflammation and tumor progression or relapsing in GBM (21). Indeed, recent data showed the ability of MET of targeting the inflammatory tumor microenvironment, contributing to reduction of tumor mass and of cancer related M2 macrophage polarization (22).

For the reasons above, the primary objective of our study was to evaluate the effect of MET used in combination with TMZ on EGFR mutation (d2-7) carrying GBM models sensitive and resistant to TMZ and on patient-derived EGFR amplified Cancer Stem Cell line. Furthermore, we aimed to evaluate the potential use of *in vivo* Positron Emission Tomography (PET) molecular imaging to predict drug effects. For this purpose we measured at early time after treatment the uptake of [^18^F]FLT and [^18^F]VC701 radiopharmaceuticals targeting thymidine kinase 1 (TK1) and Translocator Protein 18 kDa (TSPO) which are receptors associated with glioma malignancy. Despite its presence has been described also in tumors, increased levels of TSPO are associated with the presence of clusters of microglial/macrophage cells with an activated phenotype (23). For this reason, TSPO ligands, including [^18^F]VC701 are used to image the inflammatory reaction present during tumor development and the relative modulation induced by drugs (24, 25). Finally, to investigate therapy effects on tumor proliferation and inflammation markers, Ki67 and Iba1 were evaluated *post mortem* by immunohistochemistry (IHC).

## Materials and Methods

### Cell Culture

Sensitive (Gli36ΔEGFR-1 and L0627) or resistant (Gli36ΔEGFR-2) to TMZ GBM cells representative of classical subtype were used in this study. Human GBM Gli36ΔEGFR cells (kind gift of Dr. David Louis, Molecular Neurooncology Laboratory, MGH, Boston, MA) (26, 27) carry a mutant epidermal growth factor receptor (Δ2-7, EGFR). Gli36ΔEGFR cells were called Gli36ΔEGFR-1 to underline the sensitivity to Temozolomide (TMZ) treatment compared to the cell line obtained after treatment with sub-lethal doses of TMZ (50 µM of TMZ for 1 month) defined as Gli36ΔEGFR-2 (28). Cells were maintained in Dulbecco’s Modified Eagle Medium (DMEM) with high glucose supplemented with 10% heat-inactivated Foetal Bovine Serum (FBS), and 50 IU/ml Penicillin/Streptomycin (P/S), 2 mM glutamine (all Euroclone, UK) at 37°C in a 5% CO_2_/95% air atmosphere. L0627 GBM CSCs, established at the Neural Stem Cell Biology Unit, San Raffaele Scientific Institute, Milan, Italy and validated in Narayanan et al. (29) and Mazzoleni et al. (30) were cultured under the conditions of the NeuroSphere Assay (NSA) (31). GBM cells were carefully cultured and monitored and *in vitro* displayed a typical growth pattern and phenotype.

### Treatments Assay

10,000 cells/cm^2^ Gli36ΔEGFR-1 and Gli36ΔEGFR-2 cells were exposed to different concentrations of TMZ (0.1, 1, 5, 10, 25, 50, 100, 200 µM) (Sigma Aldrich, St. Louis, MO, USA) to determine the optimal dose able to distinguish TMZ sensitivity. Then, 10 mM of MET (Sigma Aldrich, St. Louis, MO, USA) alone or in combination with 25 µM of TMZ was added to the medium once at the beginning of the experiment and cell growth was monitored after 24, 48 and 72 hours (h). Cell viability was evaluated by Trypan blue exclusion test. The effect of MET, TMZ or MET plus TMZ was determined as growth inhibition rate and measured as: [1-(C_f_/C_0_)_A_/(C_f_/C_0_)_V_]*100, where C_f_ is the cell number at the point analyzed, C_0_ is the cell number at the beginning of treatment, A is the corresponding drug and V is the vehicle as previously described (12). For L0627 cells, short-term proliferation/survival studies were performed as previously described (32). Apoptosis or necrosis were assessed by Real time-Glo Annexin Apoptosis and Necrosis Assay (Promega Corporation, Madison, Italy).

### RNA Extraction and Real-Time PCR

RNA was extracted using the commercially available illustra RNAspin Mini Isolation Kit (GE Healthcare, Italy), according to manufacturer’s instructions. Total RNA was reverse-transcribed to cDNA using the High Capacity cDNA Reverse Transcription Kit (Thermo Fisher Scientifics, USA). Real-time PCR was performed in duplicate for each data point by using the Sybr Green technique and the oligonucleotides used were: β-*actin* (FRW: TCAAGATCATTGCTCCTCCTG, REV: CCAGAGGCGTACAGGGATAG); *bax* (FRW: ATG GAC GGG TCC GGG GAG; REV: ATCCAGCCCAACAGCCGC); *bad* (FRW: CCCAGAGTTTGAGCCGAGTG; REV: CCCATCCCTTCGTCGTCCT); *bcl-2* (FRW: GATTGTGGCCTTCTTTGAG, REV: CAAACTGAGCAGAGTCTTC); *sox2* (FRW: GCACATGAACGGCTGGAGCAACG; REV: TGCTGCGAGTAGGACATGCTGTAGG). Changes in the target mRNA content relative to housekeeping (*β-actin*) were determined with the ΔΔct method. Basal level expression of *sox2* gene was expressed as difference between the target mRNA content and the housekeeping (*β-actin*) (Δct).

### Animal Models and Treatment

Animal experiments were carried out in compliance with institutional guidelines for the care and the use of experimental animals, which have been authorized by the Italian Ministry of Health (n°220/2016-PR and n°378/2019-PR). Seven to eight weeks old female nude mice (Envigo RMS srl, San Pietro al Natisone, Italy) were housed at constant temperature (23°C) and relative humidity (40%) under a regular light/dark cycle, with food and water freely available. The orthotopic tumor models were obtained by the stereotactic injection of 3-5*10^5^ cells (Gli36ΔEGFR-1, Gli36ΔEGFR-1 or L0627) in 2 µl of plain DMEM with a 10 µl Hamilton syringe as previously described (10). After cells injection, mice were monitored every day for body weight and clinical signs of disease (fur, eye, motor impairment) and sacrificed at the appearance of evident signs of illness or at the loss of more than 25% of the initial body weight. Firstly, we performed a pilot study on mice inoculated with Gli36ΔEGFR-1 and with Gli36ΔEGFR-2 (n = 6 per each cell line) to monitor tumor growth with MRI at day 5 after surgery and the sensitivity to TMZ. Based on data obtained on pilot study, we decided to start drug administration 7 days after cells inoculation and perform overall survival studies. Gli36ΔEGFR-1 (R-1), Gli36ΔEGFR-2 (R-2), and L0627 tumor bearing mice were randomly assigned to 4 groups of treatment, according to the following scheme: Group A (n = 5 R-1; n = 13 R-2; n=10 L0627) received daily oral administration of Temozolomide (TMZ, 70 mg/kg) in 10% DMSO, 5 days for a 28 days cycle and repeated with this scheme (5/28) until sacrifice of animal; group B (n = 6 R-1; n = 5 R-2; n=5 L0627) received intra peritoneal (i.p.) administration of Metformin (MET, 250 mg/kg) in saline for 5d/wk for the entire treatment period; group C (n = 5 R-1; n = 6 R-2; n=8 L0627) received the combination of daily oral administration of TMZ (70 mg/kg) 5 days for a 28 days cycle and i.p. daily administration of MET (250 mg/kg); group D (n = 9 R-1; n = 9 R-2; n=10 L0627), as vehicle group, received vehicle administration (10% DMSO in saline by oral gavage and 100% saline i.p.). The treatment schedule was decided on the basis of previous studies (12) and of the dose regimen used in clinical practice adapted to mice body surface (**Figure S1**) (33). The tumor mass presence was confirmed in a subset of animals (n = 3 per treatment group) using MRI as described below. At the onset of signs of severe illness (weight loss > 25% or hemiplegia), mice were sacrificed under anesthesia and brains collected for histological analyses. Treatment efficacy was evaluated as time to sacrifice indicated as “overall survival” using the Kaplan-Meier estimator.

### Immunohistochemical Analysis

Mice brains were collected at sacrifice and fixed in 10% buffered formalin (Sigma-Aldrich) as described (34). After standard histological samples processing, serial 3 um-thick brain sections were cut and stained with hematoxylin and eosin (H&E) for morphological evaluation or probed with the following primary antibodies: Ki67 (Agilent Technologies, Santa Clara, US.), Sox2 (Cell signaling technologies Leiden, The Netherlands), cleaved-Caspase 3 (Cell Signaling Technologies, Palo Alto, CA, USA), and Iba1 (Wako pure Chemical Ind. Ltd.). Afterword, slides were incubated with Rodent Block R immunohistochemical reagent (Biocare Medical) before secondary antibody addition. Finally, slides were revealed using DAB as chromogen and counterstained with hematoxylin. Stained slides were scanned using Aperio digital scanner instrument (Leica Microsystems) and Iba1 expression was analyzed using the cytoplasmic algorithm available within ImageScope software (Leica Microsystems) after optimization of cell recognition parameters (12). Briefly, Iba1 quantification was performed by drawing regions of interest of about 0.21 mm^2^ inside the tumor, in the peripheral inner and outer part of the mass to evaluate intratumor, peritumor or distant active microglia, respectively. Ki67 marker was quantified by drawing the same regions of interest only inside the tumor. The mitotic index in each sample was scored as the average of mitotic cells per 10 High power fields (HPFs; 40X objective) on H&E slides as previously described (35). If the tumor area was smaller than 10 HPF, then the whole tumor tissue was examined for presence of mitotic cells. In supplementary table 1 and 2 mice used in the study are summarized indicating for each animal, treatment condition and day of sacrifice.

### *In Vivo* Imaging

MRI was performed with a 7T small animal magnetic resonance scanner (Bruker, BioSpec 70/30 USR, Paravision 5.1, Germany), equipped with 450mT/m gradients (slew-rate: 3400–4500T/m/s; rise-time: 140ms). A phased-array mouse-head coil with four phased-array channels was used as receiver, coupled with a 72 mm linear-volume coil as transmitter. Mice were anesthetized with isoflurane (2% in oxygen) and positioned prone on a dedicated heated apparatus, to prevent hypothermia. A coronal 2D High Resolution (HR) Rapid Acquisition with Relaxation Enhancement (RARE) T2 and a RARE T1 were acquired. After the injection of 0.2 µl/g of gadobutrol (Gadovist, Bayer Schering Pharma, Berlin-Wedding, Germany), acquisition of the RARE T1 was repeated. Tumor volume was calculated by manual contour of the post-contrast RARE T1 sequence made by an expert neuroradiologist. Gd-T1-weighted MRI was conducted to verify the presence of lesion before the beginning of treatment and for manual co-registration with PET images performed using PMOD 3.2 software.

PET imaging was performed with 3′-deoxy-3′-[^18^F]fluorothymidine ([^18^F]FLT) and [^18^F]VC701 to assess proliferation related to TK1 and TSPO receptor expression, respectively. [^18^F]FLT and [^18^F]VC701 uptake was evaluated by PET in distinct groups of Gli36ΔEGFR-1, Gli36ΔEGFR-2 and L0627 mice during control condition ([^18^F]FLT: n = 11 for R-1, n = 11 for R-2 and n = 9 for L0627; [^18^F]VC701: n = 9 for R-1, 11 for R-2 and n = 11 for L0627) and 1 week after the beginning of treatment (vehicle, TMZ, and TMZ plus MET). MET treated animals did not perform imaging. The sample size of each group is indicated in the figures.

Mice were injected *via* the tail vein with 4.18 ± 0.28 MBq of [^18^F]FLT and 4.79 ± 0.91 MBq of [^18^F]VC701. PET acquisitions were performed at 60 min ([^18^F]FLT) or 120 min ([^18^F]VC701) after tracer injection using the YAP-(S)-PET II small animal tomograph (ISE s.r.l., Pisa, Italy) or X-ß-CUBE (Molecubes, Gent, Belgium) as already described (36, 37). All the radiopharmaceuticals injected had a radiochemical purity greater than 99%. PET images were acquired in 3D mode. All the images were co-registered to MRI and quantified with PMOD 3.2 software (Zurich, Switzerland). The quality of co-registration was judged and confirmed by two independent experts in the field (S.V. and I.R.). Two different volumes of interest (VOI) were defined: (i) a control VOI covering the left striatum (volume 7 mm^3^) was drawn on the axial MR images, adjusted on the other imaging planes and then copied on the PET images of each mouse; (ii) a second glioma-covering VOI was drawn in the tumor-affected brain hemisphere and centered on mice lesions. For quantification, Standardized Uptake Value (SUV) was calculated according to the formula: SUV = tumor concentration activity [MBq/g]/(injected activity [MBq]/animal weight [g]). Maximum tracer uptake in tumor was normalized to the corresponding mean values of uptake of the contralateral control VOI (background) and indicated as tumor to background ratios (T/B ratio). A similar analysis for normal brain parenchyma was performed on normal mice.

### Statistical Analysis

*In vitro* experiments were repeated three times giving reproducible results. Data are presented as mean values ± standard deviation (SD) or standard error of the mean (SEM) of three independent experiments. For statistical analysis, non-parametric t-test, one-way analysis of variance (ANOVA), followed by Tukey’s multiple comparison test, or two-way ANOVA, followed by Bonferroni’s multiple comparison test, were performed using Prism 5 (Graph Pad Software Inc., CA, USA). Log-rank Mantel-Cox test was performed for survival comparison followed by Holm-Sidak method for multiple comparisons correction. Differences were considered statistically significant when p < 0.05.

## Results

### Combination of Temozolomide and Metformin Reduces Cell Growth and Promotes Early Apoptosis Overcoming TMZ Resistance

**Figure 1:**
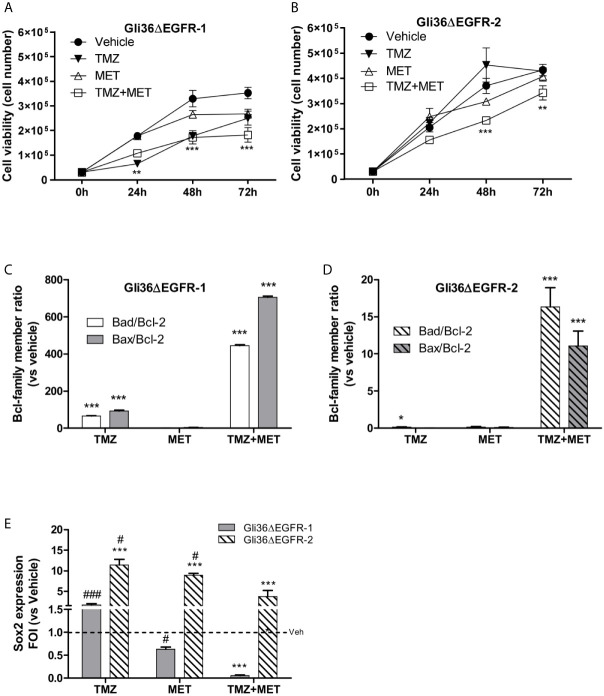
*In vitro* treatment efficacy. Evaluation of time-response viability of TMZ-sensitive **(A)** and –resistant **(B)** cells after vehicle, TMZ, MET or TMZ+MET treatment. Cell viability was assessed by means of a Trypan blue exclusion test and expressed as the number of viable cells after 24, 48 or 72 h of treatment. Two-way ANOVA analysis followed by Bonferroni’s multiple comparisons test was performed, **p < 0.01, ***p < 0.001 vs vehicle-treated cells. The induction of pro-apoptotic (*bad* and *bax*) and anti-apoptotic genes (*bcl-2*) was analyzed by means of real-time PCR in glioma Gli36ΔEGFR-1 **(C)** and Gli36ΔEGFR-2 **(D)** cells treated with TMZ, MET or the combination and expressed as *bax/bcl-2* or *bad/bcl-2* ratio. One-way ANOVA analysis followed by Tukey’s multiple comparison test was performed, *p < 0.05, **p < 0.01, ***p < 0.001. **(E)**, TMZ+MET treatment decreased *sox2* expression measured using q-real-time PCR in Gli36ΔEGFR-1 cells and counteracted its increase in Gli36ΔEGFR-2 cells. One-way ANOVA analysis followed by Tukey’s multiple comparison test was performed, ***p < 0.001 vs vehicle-treated cells; ^#^p < 0.05, ^###^p < 0.001 vs TMZ+MET treatment. The molecular data were normalized to β-actin, and the ΔΔct values were expressed as fold of induction (FOI) of the ratio between treated and control cells. Data were expressed as mean values ± SD of three independent experiments. FOI, fold of induction; MET, metformin; TMZ, temozolomide.

We identified the minimal *in vitro* dose of TMZ able to reduce Gli36ΔEGFR-1 (TMZ-sensitive cells) but not Gli36ΔEGFR-2 (TMZ resistant cells) viability. This dose was defined as 25 μM for 48 h (**Figure S2**). Then, 10 mM of MET alone or in combination with 25 µM of TMZ was added to the medium once at the beginning of the experiment and growth of Gli36ΔEGFR-1 and Gli36ΔEGFR-2 cells was monitored after 24, 48 and 72 hours. The treatment with MET alone induced a significant reduction in cell growth rate only in Gli36ΔEGFR-1 compared to vehicle (p < 0.01 at 48 h and p < 0.001 at 72 h). Furthermore, the growth rate of Gli36ΔEGFR-1 was significantly reduced after TMZ treatment at any time (p < 0.001 at 24, 48 and 72 h). The combination of TMZ and MET (TMZ+MET) displayed an additive effect at 72 h for Gli36ΔEGFR-1 (p < 0.01 vs TMZ) and reverted TMZ resistance of Gli36ΔEGFR-2 cells already after 24 h of combined treatment (p < 0.01 at 24 and 48 h and p < 0.05 at 72h) (**Figure 1A**), confirming the potential efficacy of MET in reducing cell resistance to TMZ (**Figure 1B**). The combination of TMZ+MET significantly reduced Gli36ΔEGFR-1 cell growth also compared to MET alone (p < 0.01 at 24 h, p < 0.001 at 48 and 72 h). These results were confirmed in a patient-derived Cancer Stem Cell (CSC) line (L0627) that shows features typical of the classic molecular subgroup, such as the overexpression of EGFR gene (30). Also in this case, the combination of TMZ+MET significantly decreased cells survival compared to single therapy at 72 h (p = 0.004 vs TMZ and p = 0.012 vs MET) (**Figure S3**).

In line with the observed effect on cell viability, the association of TMZ and MET treatments deregulated the balance between pro-survival (*bcl2*) and pro-apoptotic (*bax/bad*) Bcl-family members in both Gli36ΔEGFR-1 and Gli36ΔEGFR-2 cells (**Figures 1C, D**) as indicated by the increase of *bad/bcl-2* and *bad/bcl-2* ratios. On the contrary, TMZ alone increased *bad/bcl-2* mRNA expression only in Gli36ΔEGFR-1 cells and to a lower extent compared with TMZ+MET treatment. To evaluate the apoptosis induced by treatments, cells were assessed for exposure of Phosphatidylserine (PS) on the outer leaflet of the cell membrane. Supplementary figure 4 showed that only TMZ in Gli36EGFR-1 displayed secondary necrosis with PS translocation to the outer leaflet and loss of membrane integrity. This result suggested that MET and TMZ+MET showed early apoptosis characterized by PS translocation to outer leaflet but no cell membrane disruption (**Figure S4**).

### SOX2 Expression Is Hampered by MET Addition to TMZ

SOX2 has been reported to play a pivotal role in developing drug resistance of glioma (11, 38) and its expression was correlated with the grade of malignancy and favored the maintenance of an undifferentiated state of cancer stem cells (39, 40). Furthermore, recently its role as glioma stem cell biomarker has been described in association with NANOG and OCT (41, 42).

We performed qRT-PCR to investigate if the different treatments could modulate *sox2* expression in TMZ-sensitive and -resistant cells. The basal *sox2* mRNA level was similar in Gli36ΔEGFR-1 and Gli36ΔEGFR-2 cells (0.082 and 0.079 respectively). Single-drug treatments did not alter *sox2* expression in Gli36dEGFR-1 cells. In contrast, the combination of the two drugs significantly reduced *sox2* (p = 0.0005) levels (**Figure 1E**). On the contrary, in Gli36ΔEGFR-2 all treatments led to a dramatic increase of *sox2* expression. However, the increment of *sox2* transcript was significantly less pronounced with the combinatorial treatment (**Figure 1E**). These findings suggest that in TMZ-sensitive cells, the chemotherapeutic perturbed cell viability and partially reduced the determinants of therapy resistance. In this system, the addition of MET was synergistic. On the contrary, TMZ-resistant cells showed an enhanced multi-drug resistant phenotype and MET administration was only able to partly reduce TMZ-induced *sox2* overexpression. *sox2* is also expressed on L0627 cell (personal communication from R. Galli) but the effects of drugs were not evaluated.

### TMZ Plus MET Protocol Increases Survival of Orthotopic GBM Mouse Models

In a pilot study, after 5 days from cell injection, all animals were positive for intracerebral lesions at MRI (tumor volume: 4.38 ± 1.67 mm^3^ and 6.12 ± 2.11 mm^3^ for Gli36ΔEGFR-1 and Gli36ΔEGFR-2 respectively). In the same study we confirmed *in vivo* the different sensitivity of Gli36ΔEGFR-1 and Gli36ΔEGFR-2 to TMZ (% tumor increase after treatment: 22.7 vs 54.9).

**Figure 2:**
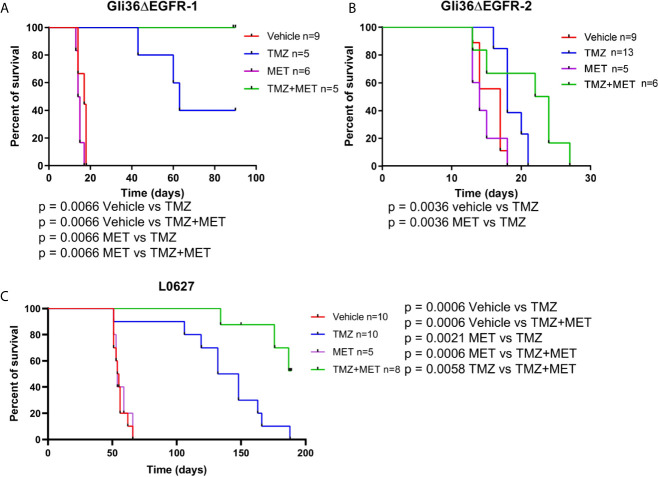
Survival curves of GBM-bearing mice. **(A–C)**, nude mice were injected with Gli36ΔEGFR-1, Gli36ΔEGFR-2 or L0627 cells. At tumor onset, mice were grouped by different treatments and treated with vehicle, TMZ, MET or TMZ+MET (n = 13-5/group as indicated). Log-rank Mantel-Cox test was performed for survival comparison followed by Holm-Sidak method for multiple comparisons correction. MET, metformin; TMZ, temozolomide.

Then, we analyzed the efficacy of MET and TMZ treatments alone or in combination in orthotopic mouse models of glioma obtained by Gli36ΔEGFR-1 or Gli36ΔEGFR-2 cells inoculation. The MET protocol alone did not show any effect on survival of both GBM models. As expected, in the TMZ-sensitive Gli36ΔEGFR-1 model, TMZ treatment significantly increased mice overall survival compared to animals treated with vehicle (median survival 63 days vs 17 days, p = 0.0066). Among TMZ-treated mice only one mouse was histologically confirmed as disease-free, while others mice showed tumor relapse detected by MRI or IHC (**Figures S5A, B**). More interestingly, the combination of TMZ+MET extended the overall survival of Gli36ΔEGFR-1 mice up to 90 days, when we decided to sacrifice all mice which were histologically confirmed as disease-free (**Figure 2A**). In mice bearing TMZ-resistant glioma, TMZ alone did not prolong mice survival compared with that of vehicle-treated mice (18 days vs 17 days). In this case, TMZ+MET protocol slightly increased median survival time up to 23 days (**Figure 2B**). EGFR-2 bearing mice regardless of treatment displayed tumor increase at MRI after one week (**Figures S5C, D**).

When CSC line L0627 was tested in the same settings, orthotopically transplanted mice displayed tumor masses at later times (tumor onset at approximately 40 days) in comparison with Gli36ΔEGFR mice. Confirming our previous data, also in the L0627 model, treatment with MET alone did not show any therapeutic efficacy (median survival 54.5 days and 54 days for control and MET, respectively) but, most remarkably, a significant increase of survival rate could be detected in animals treated with the MET+TMZ combination in comparison with TMZ alone group (p = 0.0058) (**Figure 2C**). In addition, after an initial response, all mice treated with TMZ showed tumor relapse at the MRI, whereas only 3 out of 8 TMZ+MET treated animals developed tumor recurrence (**Figures S6A**, **B**).

### Analysis of Glioma Proliferation Index and Inflammation After Treatment Reveals Superior Anti-Tumor Efficacy of TMZ Plus MET Protocol in Mouse Models of Glioma

**Figure 3:**
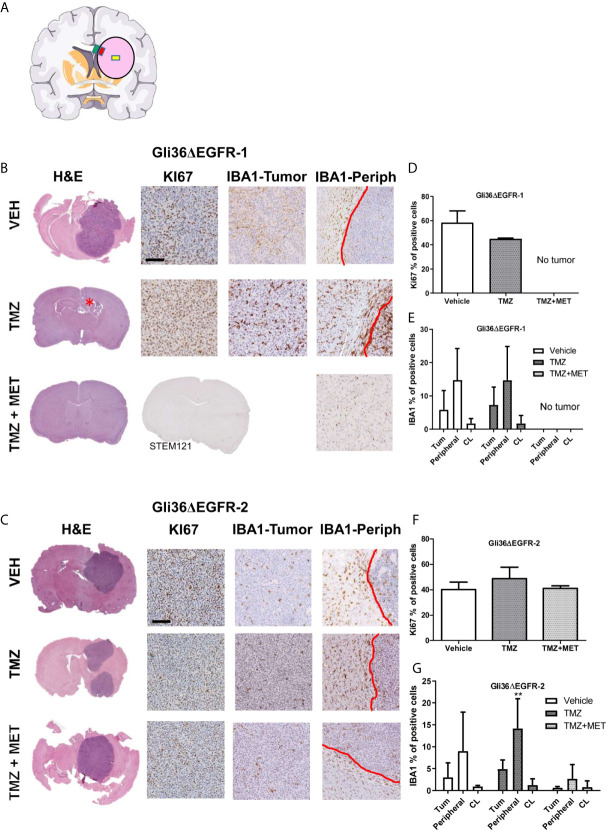
Evaluation of KI67 and IBA1 in GBM mouse samples at the end of survival study. **(A)**, schematic representation of the brain areas sampled for Iba1 quantification. The pink circle indicates the tumors, the yellow square indicates the Regions of Interest (ROI) placed on the tumor, the red one that placed on the inner border, and the green one that placed on the outer border. **(B, C)**, images of representative hematoxylin and eosin (H&E), Ki67 and Iba1 staining for intracranial Gli36ΔEGFR-1 and Gli36ΔEGFR-2 tumors from mice treated with vehicle (VEH, n=5 for R1, n=3 for R2), TMZ (n=2 for R1, n=4 for R2) and TMZ+MET (n=5 for R1 and n=3 for R2) euthanized at the end of survival study. Gli36ΔEGFR-1 TMZ+MET tumors were stained also with STEM121. **(D, F)**, quantification of the ki67 marker expressed as the percentage of positive cells. One-way ANOVA analysis followed by Tukey’s multiple comparison test. **(E, G)**, quantification of IBA1 marker expressed as percentage of positive cells in the inner part of the tumor (Tum), in tumor-brain border (Peripheral) and in the brain region contralateral to the tumor (CL). Two-way ANOVA analysis followed by Bonferroni’s multiple comparison test, **p = 0.0050 TMZ+MET vs TMZ.

To gain insights into *in vivo* effects of the different therapeutic protocols, we evaluated glioma proliferation index and inflammation (i.e. microglia/macrophage activation) in mouse brains collected after cancer-related death or sacrifice. Accordingly, Ki67 and Iba1 staining were performed and quantified in different brain areas (**Figure 3A**).

Gli36ΔEGFR-1-tumors treated with vehicle showed a mean proliferation index of 58.4%. In the TMZ arm one mouse did not display tumor mass whereas the two tumors processed displayed a proliferation index of 44.4% and 45.3%. No tumor mass was detected in all samples from the TMZ+MET arm (n = 5). For this reason, we also performed STEM121 staining, a marker able to identify human cells in a mouse tissue, which confirmed the absence of human cells. Gli36ΔEGFR-2 tumors from vehicle-treated animals had a mean proliferation index of 40.1%, whereas those from the TMZ or the TMZ plus MET arms showed a mean Ki67 staining of 48.6% and 40.9%, respectively (**Figures 3B–D, F**).

When we looked at microglia/macrophage activation, we did not observe any difference between vehicle and TMZ-treated Gli36ΔEGFR-1 tumors (**Figure 3E**); after treatment with TMZ plus MET no signs of tumor or Iba1 markers were visible. In Gli36ΔEGFR-2 generated glioma, TMZ+MET significantly reduced infiltration of Iba1 positive myeloid cells only at the tumor-brain border compared to TMZ (p = 0.005 versus TMZ, **Figure 3G**). On the other areas data are very heterogeneous.

For the L0627-generated tumors we scored the mitotic count in three high power fields per mouse and tumor cell viability using an antibody toward cleaved-caspase 3, because Ki67 staining was surprisingly absent.

This analysis showed that tumor proliferation was decreased by TMZ or MET individual treatments (20.88 for vehicle, 14.38 for TMZ, 13.90 for MET), but only TMZ marginally increased glioma cells apoptosis (0.68% for vehicle, 4.35% for TMZ and 2.56% for MET) (**Figures S7A, C**). Again, the combinatorial treatment significantly impaired the ability of the glioma cells to *in vivo* grow, one mouse negative-defined at MRI showed a small tumor mass at sacrifice. In regards of the glioma stem cell markers, we observed a high Nestin staining in all L0627 tumor samples and positive staining for Iba1 within and in the peripheral areas of the tumor and in cerebral hemisphere contralateral to tumor implantation (**Figure S7B**). Interestingly, both MET+TMZ-treated analyzed mice (both that with a small tumor mass and that disease-free) displayed Iba1-positive staining only in the cerebral hemisphere contralateral to tumor implantation indicating an activation of macrophages/microglia.

### Post-Treatment [^18^F]FLT-PET Uptake Is a Measure of TMZ Responsiveness in the Gli36ΔEGFR Model

**Figure 4:**
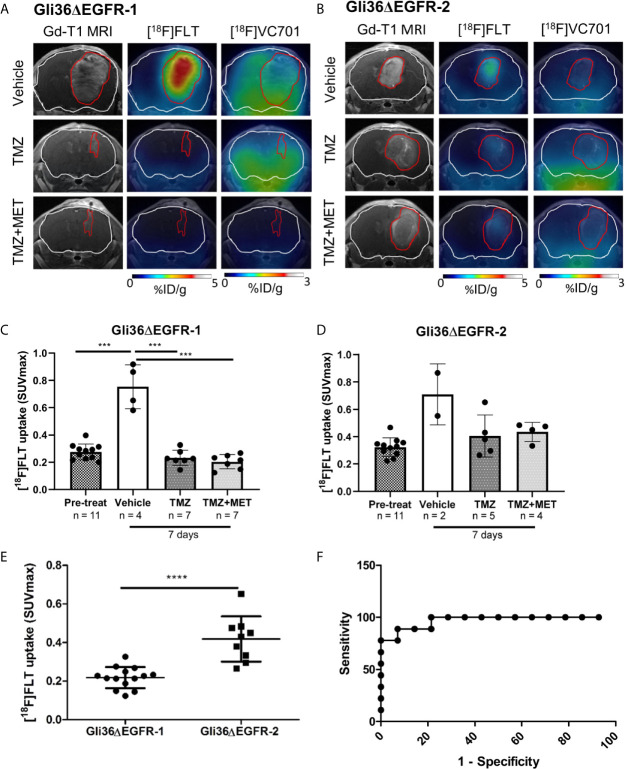
[^18^F]FLT uptake distinguished between TMZ-sensitive and –resistant tumors. Representative Gadolinium-T1w MRI images and their fusion with PET images for [^18^F]FLT and [^18^F]VC701 of Gli36ΔEGFR-1 **(A)** and Gli36ΔEGFR-2 tumor-bearing mice **(B)** after 7 days of vehicle, TMZ, and TMZ+MET treatment. The white line indicates the brain and the red one indicates the tumor area depicted by MRI. [^18^F]FLT uptake expressed as SUVmax in Gli36ΔEGFR-1 **(C)** and in Gli36ΔEGFR-2 **(D)** tumor-bearing mice before and after 7 days of treatment. One-way ANOVA analysis followed by Tukey’s multiple comparison test was performed, ***p < 0.001. In the bottom part of both graphs, the sample size for each group is indicated. **(E)**, After 7 days of treatment with TMZ or TMZ+MET, mice with TMZ-resistant tumors showed a significant increase of [^18^F]FLT uptake compared to TMZ-sensitive ones. Unpaired t-test analysis was performed, ****p < 0.0001. **(F)**, ROC analysis of [^18^F]FLT SUVmax for prediction of different response to TMZ therapy. Optimal cut-off point was defined for [^18^F]FLT as 0.3295 (77.8% sensitivity; 100% specificity).

We used PET with [^18^F]FLT to determine if this tracer could identify responder from non-responder mice after treatment. Gli36ΔEGFR-1 and Gli36ΔEGFR-2 generated tumors displayed similar [^18^F]FLT pre-therapy uptake values, i.e. a SUVmax value of 0.28 ± 0.06 and of 0.32 ± 0.07, respectively (**Figures 4A–D**).

Early after therapy start (day 7), [^18^F]FLT uptake increased in all vehicle-treated mice (either carrying a Gli36ΔEGFR-1 or Gli36ΔEGFR-2 tumors) compared to basal levels (p < 0.001; **Figure 4C**). All treatment protocols significantly reduced the increase of [^18^F]FLT uptake both in Gli36ΔEGFR-1 bearing mice (p < 0.001 versus vehicle; **Figures 4A, C**) and in Gli36ΔEGFR-2 glioma, although to a lower extent (**Figures 4B, D**).

We successfully identified a cut-off value of [^18^F]FLT PET-SUVmax able to discriminate TMZ-responder (considering both TMZ- and TMZ+MET-treated animals) from therapy-resistant tumors. Appling Receiver Operating Characteristic (ROC) curve to post-treatment [^18^F]FLT PET data, we identified a threshold value of SUVmax (corresponding to 0.3295) with a sensitivity and specificity of 77.8% and 100%, respectively, in distinguishing animals response to treatments (**Figures 4E, F**). Therefore our data suggest that [^18^F]FLT-PET may be used to monitor treatment responses in glioma and to identify patients as responders or not after treatment. Unfortunately, [^18^F]FLT PET was not able to predict response duration.

This study was repeated also in the CSC-transplanted mice. In agreement with the results on Ki67, we didn’t observe any significant uptake of [^18^F]FLT in L0627 tumors independently from the lesions’ dimension or the treatments’ protocol, possibly due to the lowly proliferative, highly infiltrative nature of CSCs and to the maintenance of Blood Brain Barrier (BBB) integrity in this specific GBM model (43).

### The Uptake of the Inflammatory-Related [^18^F]VC701-PET Probe Was Reduced by TMZ Plus MET Treatment in Gli36ΔEGFR-1 Tumors and Negatively Associated With Treatment Response

**Figure 5:**
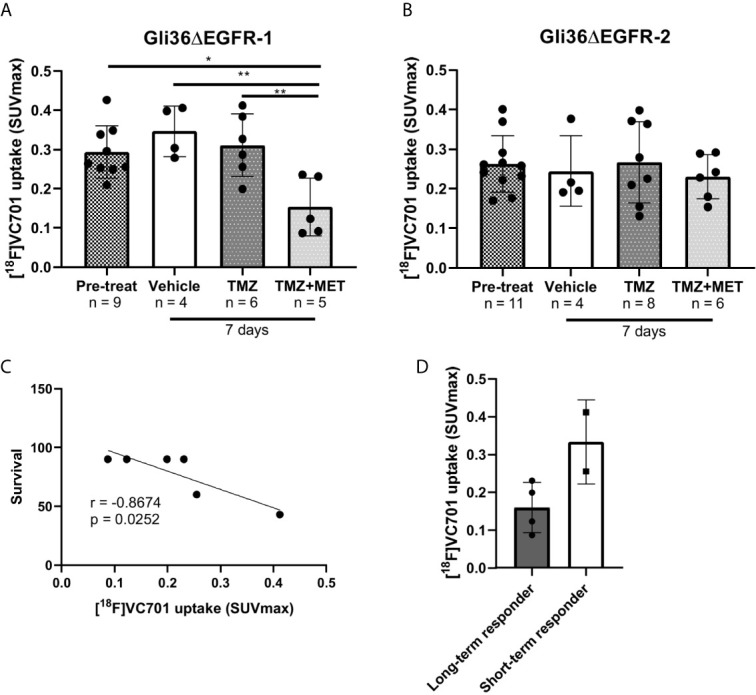
[^18^F]VC701 uptake in GBM mouse models. **(A)**, [^18^F]VC701 uptake expressed as SUVmax in Gli36ΔEGFR-1 tumor-bearing mice decreased after 7 days treatment with TMZ+MET. One-way ANOVA analysis followed by Tukey’s multiple comparison test. *p < 0.05, **p < 0.01. **(B)**, No changes of [^18^F]VC701 uptake was observed in Gli36ΔEGFR-2 tumor-bearing mice. **(C)** Correlation curve between [^18^F]VC701 uptake and survival in TMZ-responder Gli36ΔEGFR-1 mice (r = -0.8674, p = 0.0252). **(D)** Graph of [^18^F]VC701 uptake expressed as SUVmax of Gli36ΔEGFR-1 long-term (disease-free mice at 90 days independently by type of treatment) and short-term survived mice. Unpaired t-test analysis was performed.

After 7 days from the beginning of treatment, vehicle- or TMZ-treated Gli36ΔEGFR-1 glioma mice displayed the same uptake of [^18^F]VC701 of the pre-treatment mice. On the contrary, the TMZ plus MET protocol in Gli36ΔEGFR-1 glioma led to a significant decrease of [^18^F]VC701 uptake (0.15 ± 0.07 SUVmax) not only in comparison with mice treated with vehicle (0.35 ± 0.07, p < 0.01) or TMZ alone (0.31 ± 0.08, p < 0.01) but also in comparison with pre-treatment uptake (0.29 ± 0.07, p < 0.05) (**Figures 4B**, **5A**). Moreover, in this model we observed an increased uptake of [^18^F]VC701 in brain regions contralateral to tumor mass regardless of treatment conditions compared to that of healthy brains (p < 0.05), except animals treated with TMZ plus MET (SUVmax values: pre-treatment = 0.27 ± 0.05; vehicle = 0.28 ± 0.06; TMZ = 0.29 ± 0.08; MET plus TMZ = 0.14 ± 0.07 and healthy brain = 0.16 ± 0.05) (**Figures S8A, B**). In Gli36ΔEGFR-1 samples, the expression of Iba1 staining was very heterogeneous in the cerebral hemisphere contralateral to tumor implantation (**Figure 3B**) and it was correlated to expression of Iba1 in outer border of tumor (R2 = 0.68, p = 0.02). No expression of Ki67 proliferation marker was detected in these areas. The increased uptake observed in the normal brain parenchyma of tumor bearing animals suggests that Gli36ΔEGFR-1 tumors enhance the inflammation status of the whole brain, an effect that is blocked by the combination of MET to TMZ. Interestingly, within the TMZ-responder Gli36ΔEGFR-1 group (TMZ- and TMZ+MET-treated animals) identified with [^18^F]FLT PET we observed a significant inverse correlation between [^18^F]VC701 uptake and survival (r = -0.8674, p = 0.0252) with long-term survived mice (disease-free mice at 90 days independently by type of treatment) displaying lower [^18^F]VC701 uptake values (**Figures 5C, D**).

On the contrary, in Gli36ΔEGFR-2 tumor bearing mice, [^18^F]VC701 uptake was lower in comparison with Gli36ΔEGFR-1 bearing mice and not affected by treatment (**Figure 5B** and **Figures S8A, B**).

Also in this case, L0627 tumors displayed a different behavior. We observed a slight but significant decrease of [^18^F]VC701 uptake in the tumor after 7 days of treatment with TMZ plus MET in comparison with vehicle animals and no significant modifications in the contralateral part of the brain both in untreated and treated mice (**Figure S9**).

## Discussion

We and others reported that MET exerts a synergic action with TMZ and it is able to revert TMZ resistance in GBM models (11, 12, 44, 45). In this study, we further evaluated the effect of MET used in combination with TMZ on two genetically homogeneous GBM models either sensitive (Gli36ΔEGFR-1) or resistant (Gli36ΔEGFR-2) to TMZ and carrying EGFRvIII mutation, representative of the classical molecular subgroup. Afterwards, we took advantage of a patient-derived Cancer Stem Cell (CSC) line (L0627) that shows features typical of the classic molecular phenotype such as the overexpression of EGFR gene to validate the results also in a preclinical model closer to clinic.

MET increases the effect of TMZ on tumor growth arrest and apoptotic markers. However, the effect of MET add-on was higher for the TMZ sensitive cell line. The differences observed may be related to the significant reduction of *sox2* levels induced by TMZ plus MET only on Gli36ΔEGFR-1. *sox2* is a typical marker of poor differentiated cells that have been associated with CSC and tumor aggressiveness whose levels are regulated by several pathways including EGFR (39, 41, 42). Indeed, high levels of *sox2* are described in presence of EGFRvIII mutation as is the case of our cell lines. Moreover, modulation of *sox2* is able to oppositely increase or reduce TMZ sensitivity (38, 46). In Gli36ΔEGFR-1, MET and particularly MET plus TMZ showed a decrease of *sox2* when compared to TMZ given alone. This effect was not observed in Gli36ΔEGFR-2 where a marked increase in *sox2* was detected with all treatments although its increase was lower when MET was associated with TMZ. An effect of MET on *sox2* concerning cell viability and animal survival on TMZ resistant cell models was previously described by Yang et al. (11). However, in their case cells were repeatedly exposed to MET prior to animal inoculation.

During *in vivo* studies, TMZ administration increased animal survival only in the sensitive cell line model. As previously observed on U251 and T98 cells, MET given alone in doses closed to those used for diabetes treatment failed to increase animal survival independently of cell line model (12). In agreement with *in vitro* results, MET potentiates the effect of TMZ. However, also in this case, the effect was higher in animals carrying Gli36ΔEGFR-1 cells. Overall survival studies were supported by post mortem IHC analysis. Gli36ΔEGFR-2-bearing mice revealed high levels of Ki67 independently on treatment schedule, confirming that in the case of EGFR mutated TMZ-resistant cells, MET is not able to efficiently counteract TMZ resistance. Treatment modulation on cell proliferation was confirmed also by PET study with [^18^F]FLT. Indeed, [^18^F]FLT uptake was reduced at early times by TMZ or TMZ plus MET treatment in mice bearing Gli36ΔEGFR-1 and post-treatment [^18^F]FLT SUV values were able to non-invasively quantify the extent of response in responder from non-responder subjects. Unfortunately, using [^18^F]FLT PET was not possible to differentiate long-term responder mice from short-term responder mice. Results on *in vitro* and *in vivo* cell growth and animal survival were confirmed on L0627, indicating that MET is able to increase TMZ effect also on CSC lines and CSC-derived xenografts. Interestingly, both in L0627 and Gli36ΔEGFR-1 model, longitudinal MRI studies showed that TMZ administration reduced or eliminated lesions after one week (**Figures S5A, B** and **S6**). However, at variable times after the beginning of treatment, we observed lesion relapse in all animals treated with TMZ alone. This effect was absent or definitely lower when MET was added to TMZ suggesting that the combination treatment is able to prevent some mechanisms associated with TMZ-induced tumor resistance. A low level of [^18^F]FLT uptake was observed in L0627 mice precluding the use of [^18^F]FLT-PET as early predictor. Kinetics modelling evidences suggest that in brain tumors, [^18^F]FLT uptake is tightly dependent on factors related to influx (BBB integrity, permeability and transport expression) and not only to thymidine kinase-1 activity (47). The low proliferative, highly infiltrative nature of CSCs in general and of these cells in particular and the maintenance of BBB integrity in this L0267 GBM model could partly contribute to the lower uptake of [^18^F]FLT observed (43).

Several evidences indicate that, in glioma, the inflammatory component increases in parallel with tumor aggressiveness and grade (48). Furthermore, glioma cells are able to recruit glioma associated peripheral monocyte/macrophage and activated microglia (GAMMs) favoring a tumorigenic phenotype (49, 50) which plays a central role in drug resistance and treatment failures. Recent findings suggest that MET is able to target the inflammatory component associated with the cancer microenvironment (22, 51). In both Gli36ΔEGFR models, we found positive Iba1 staining particularly in TMZ sensitive lesions, distributed with a decreasing gradient from outer border of the tumor to the inner regions. In Gli36ΔEGFR-1 and in L0627models, sparse Iba1 signal was also present in the brain parenchyma contralateral to tumor. This data is in agreement with what recently reported by Foray et al. (52). The authors found in Gli36ΔEGFR-1 model an increased number of peri-tumoral GAMMs and astrocytes expressing TSPO and an augmented expression of M2 phenotype myeloid markers in TMZ-treated animals. Gabrusiewicz et al., in a GL261 glioma mice model, described the presence of microglia/macrophage infiltrating cells already at 5 days after cell implantation also in the contralateral, non-tumors containing hemispheres (53) and Guardia Clausi et al. observed modifications of inflammatory cells not only in the tumor but also in the cerebral hemisphere contralateral to tumor implantation after therapy (54). In some types of cancer, MET is able to switch macrophage polarization versus M1 phenotype and this effect might participate to the capability of MET to counteract TMZ recurrences (55). Our results of TSPO PET studies performed at early time showed that [^18^F]VC701 was taken up particularly by Gli36ΔEGFR-1 GBM. High radioactivity uptake was observed not only in tumors but also in extra tumor regions in comparison with normal healthy mice. In addition, administration of MET plus TMZ was the only treatment able to reduce the [^18^F]VC701 signal in brain regions surrounding the tumors as well as in normal brain parenchyma and extra-cerebral tissues of Gli36ΔEGFR-1 but not in that of Gli36ΔEGFR-2 nor L0627 suggesting that the inflammatory signal is cell line-specific as well as their treatment-induced modulation. On the contrary to [^18^F]FLT, in TMZ-responder Gli36ΔEGFR-1 group (TMZ- and TMZ+MET-treated animals) we observed a significant inverse correlation between [^18^F]VC701 uptake and survival. Long-term survived mice (disease-free mice at 90 days independently by type of treatment) displayed lower [^18^F]VC701 uptake values. Although [^18^F]FLT signal aligns with therapy response monitored with MRI, [^18^F]FLT gives information about a biological feature of the tumor and [^18^F]FLT uptake area not necessarily overlapped with tumor volume by identifying specific areas with higher cell proliferation (56). Moreover, the combination of two radiotracers could allow to obtain complementary information to better monitor therapy response.

Firstly used to image glioma by Junck et al. (57), post-surgery studies showed that TSPO receptors are localized on tumors cells particularly in high grade glioma (58, 59). Moreover, using IHC, Su et al. demonstrated that a relevant percentage of glioma associated macrophage is negative for TSPO (60). These findings were reported also by preclinical studies on orthotopic mouse models (including the model described in the present study) showing that both tumors and microglia/macrophage components contribute to TSPO related PET signal (56). The TSPO role in GBM tumors cells is not clear. Fu et al. showed that absence rather than the increase of TSPO favors an aggressive phenotype of tumors with increased proliferation rate, glycolytic metabolism and poor outcome (61). A better knowledge of the role and the expression of TSPO is surely fundamental to use the TSPO-imaging in clinical practice but this method can be useful to monitor reactive cell infiltration.

Overall, our data confirm the lack of effect of MET given alone reported in our previous work. In addition, we showed that the association between MET and TMZ increases the treatment efficacy in mouse GBM models with EGFR-mutated or -amplified not previously exposed to TMZ. Finally, as indicated by longitudinal studies with MRI, MET association reduces the rate of recurrence during TMZ treatment. However, in a recent retrospective cohort study, Seliger et al. observed that the use of metformin was associated with better overall and progression-free survival of patients with WHO grade III associated with IDH1 mutations (62). For this reason, our results need to be confirmed in other GBM models particularly associating radiotherapy to pharmacological treatment. In our study, PET imaging with [^18^F]FLT suggests that the reduction of cell proliferation represents a common mechanism of response for TMZ whereas only TMZ plus MET was able to decrease the *in vivo* binding of [^18^F]VC701 to TSPO receptors, an effect that was associated with animals survival. These results suggest that TSPO-ligand may be used as a complementary molecular imaging marker to predict tumor microenvironment related treatment effects.

## Data Availability Statement

The raw data supporting the conclusions of this article will be made available by the authors, without undue reservation.

## Ethics Statement

The animal study was reviewed and approved by Ethics Committee of IRCCS San Raffaele Scientific Institute of Milan and Italian Ministry of Health (n°220/2016-PR and n°378/2019-PR).

## Author Contributions

SV and RM designed the study. SV, ALD, ST, BZ, ED, AC, LP, TV, VV, PR, and VP developed the methodology. SV, ALD, IR, CM, VP, and VV performed research and data analysis. SV, ALD, AJ, VP, RG, LO, VV, and RM drafted the manuscript. All authors contributed to the article and approved the submitted version.

## Funding

This research was funded by Associazione Italiana Ricerca sul Cancro (AIRC) IG 2014 grant n. 15771 and IG 2018 grant n. 21635 (RM) and by grants from the SysBioNet project, a MIUR initiative for the Italian Roadmap of European Strategy Forum on Research Infrastructures (ESFRI). The Italian Ministry for Education and Research (MIUR) is gratefully acknowledged for yearly FOE funding to the Euro-BioImaging Multi-Modal Molecular Imaging Italian Node (MMMI).

## Conflict of Interest

The authors declare that the research was conducted in the absence of any commercial or financial relationships that could be construed as a potential conflict of interest.
