# Supplementary material for: Imaging Metformin Efficacy as Add-On Therapy in Cells and Mouse Models of Human EGFR Glioblastoma
Source: Front Oncol. 2021 May 3;11:664149. doi: 10.3389/fonc.2021.664149 (PMC8126706; doi:10.3389/fonc.2021.664149)
Supplement: Supplementary file 1 [file DataSheet_1.docx]

Supplementary Material

## Supplementary Figures

**Figure S1 Schematic diagram of experimental plan**


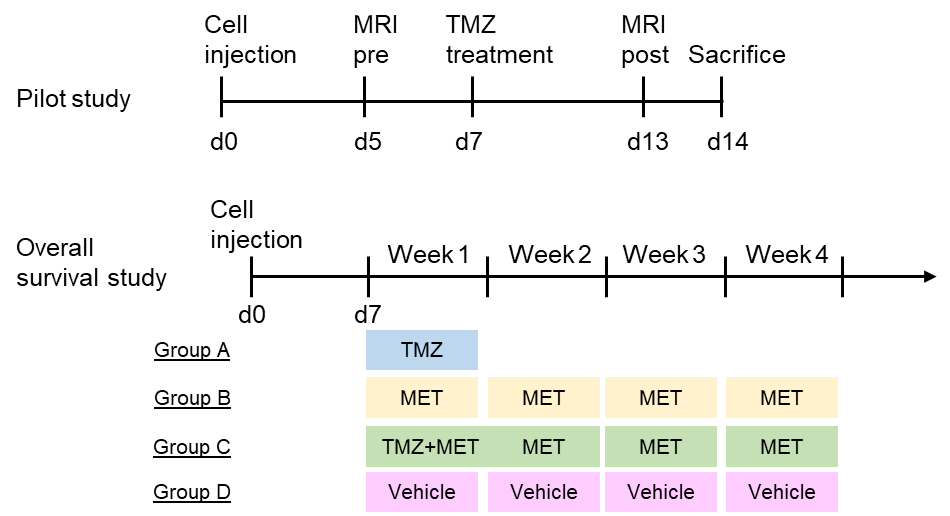


**Supplementary Figure 1.** In the pilot study, after 5 days from cell injection tumor bearing animals performed a MRI study to visualize tumor. After that, mice were treated with TMZ and after one week tumor response was evaluated using MRI to validate the different sensitivity to TMZ of the two cell lines. On the basis of this results, overall survival studies were performed. Tumor bearing animals were divided into 4 groups and treated with: group A TMZ (70 mg/kg, o.g.) in 10% DMSO, 5 days for a 28 days cycle, group B Metformin (MET, 250 mg/kg, i.p.) in saline for 5d/wk for the entire treatment period, group C TMZ+MET) received the combination of daily oral administration of TMZ (70 mg/kg) days for a 28 days cycle and i.p. daily administration of MET (250 mg/kg) and group D received vehicle administration (10 % DMSO in saline by oral gavage and 100% saline i.p.). For animal found healthy and with reduced/not detectable tumor after the first cycle, the schedule treatment was repeated until the onset of tumor recurrence or sacrifice of the animal to perform IHC analysis.

**Figure S2 Temozolomide dose-response curve**


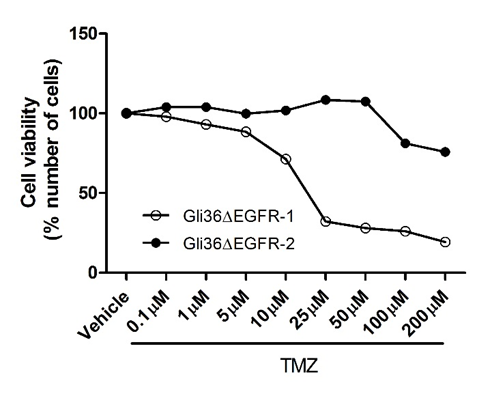


**Supplementary Figure 2.** Gli36ΔEGFR-1 and Gli36ΔEGFR-2 cells were treated with increasing doses of TMZ (0–200 μM) for 48 hours. Cell viability was assessed by Trypan blue exclusion test and expressed as % number of cells.

**Figure S3 In vitro efficacy of treatments in a GSC line**


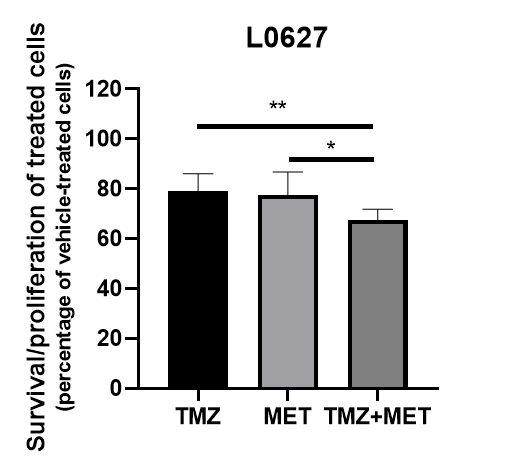


**Supplementary Figure 3.** L0627 cells were treated with 100 µM TMZ, 5 mM MET or the combination of both for 72 hours. * p = 0.0118; ** p = 0.0035. One-way ANOVA analysis followed by Tukey’s multiple comparisons test was performed.

**Figure S4 *In vitro* assessment of early apoptosis or necrosis after treatments**


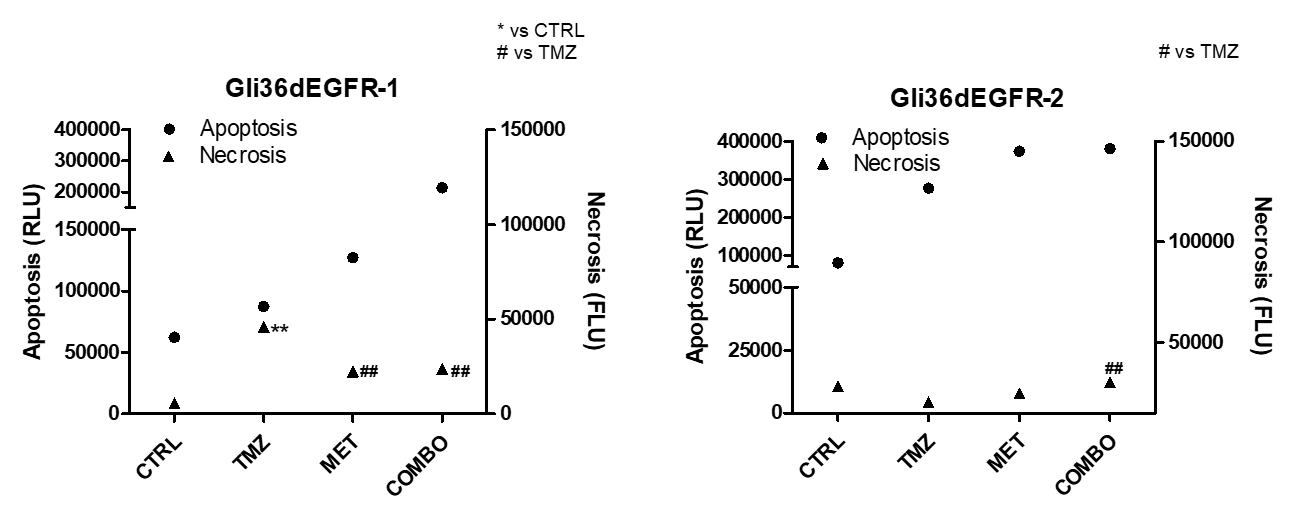


**Supplementary Figure 4.** Gli36ΔEGFR-1and Gli36ΔEGFR-2 48h after treatments were assessed for measuring early apoptosis (black dots) or secondary necrosis (black triangles). One-way ANOVA analysis followed by Tukey’s multiple comparison test was performed; Gli36ΔEGFR-1 ** p = 0.01 CTRL vs TMZ, ## p = 0.01 TMZ vs MET and TMZ vs COMBO; Gli36ΔEGFR-2 ## p = 0.03 TMZ vs COMBO.

**Figure S5 Gli36ΔEGFR-1 and Gli36ΔEGFR-2 tumor progression
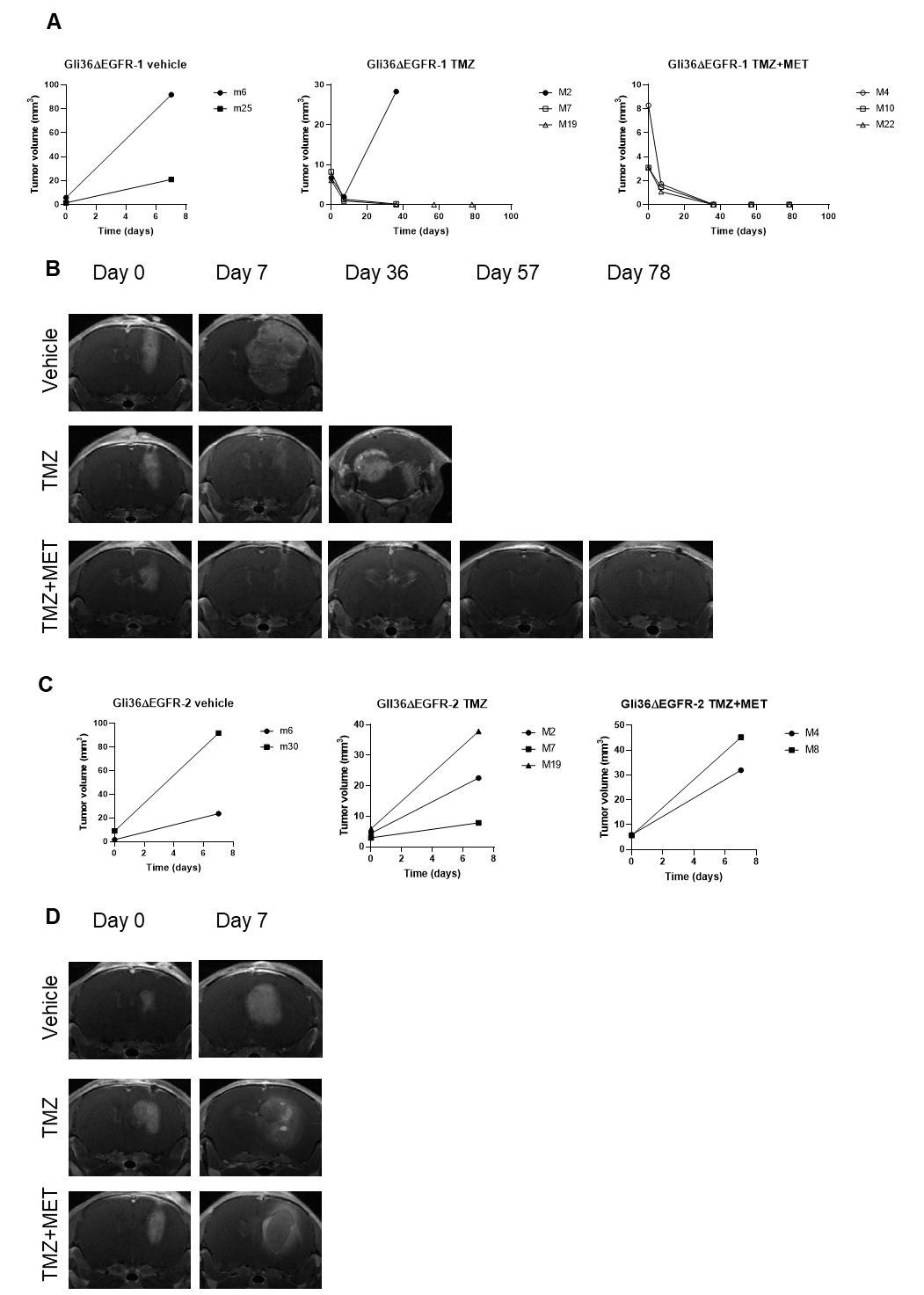
**

**Supplementary Figure 5.** A) Gli36ΔEGFR-1 tumor volume measured at different times using Gadolinium T1-weighted MRI. B) Representative tumor MRI images of Gli36ΔEGFR-1 vehicle, TMZ and TMZ+MET treated mice. C) Gli36ΔEGFR-2 tumor volume measured at different times using Gadolinium T1-weighted MRI. B) Representative tumor MRI images of Gli36ΔEGFR-2 vehicle, TMZ and TMZ+MET treated mice.

**Figure S6 L0627 tumor progression**

**
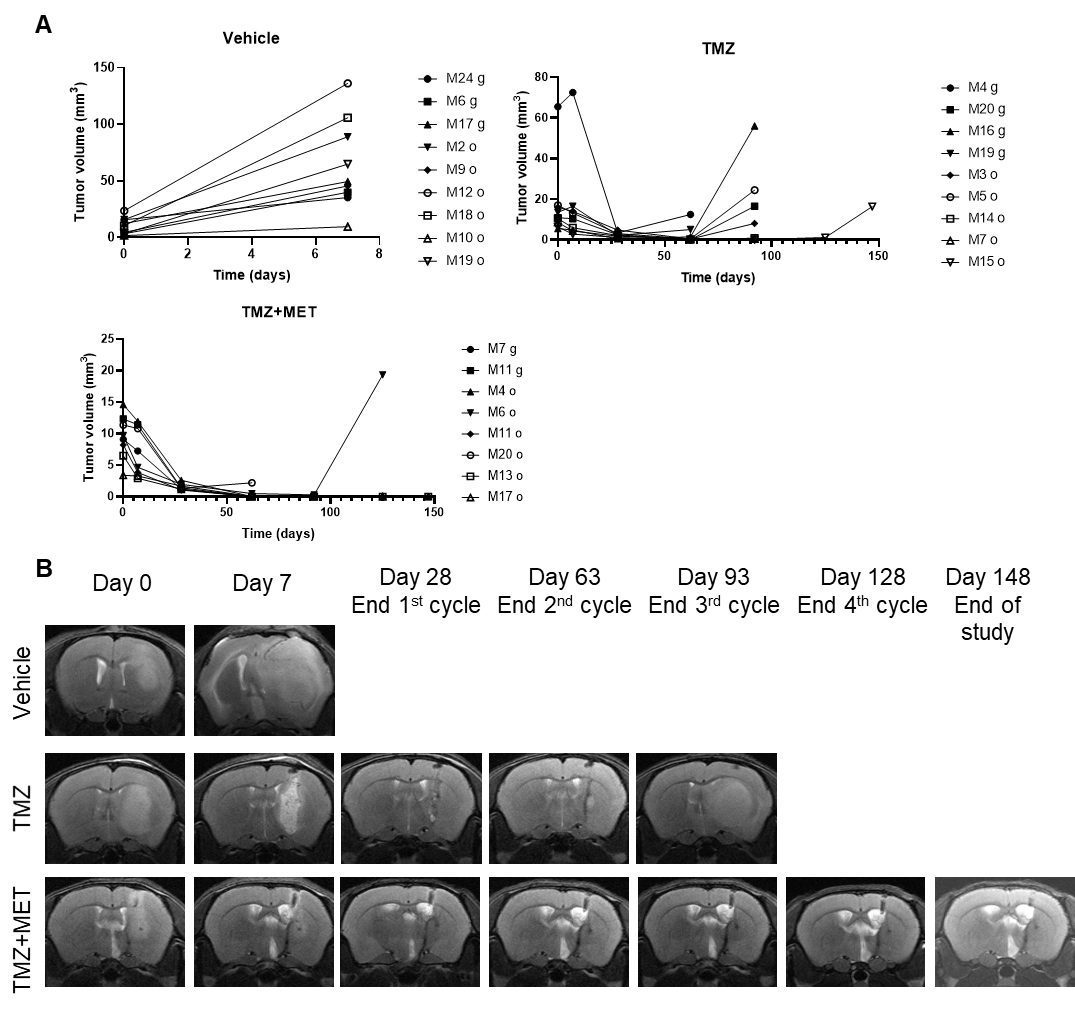
**

**Supplementary Figure 6.** A) Tumor volume measured at different times using T2-weighted MRI. B) Representative tumor MRI images of vehicle, TMZ and TMZ+MET treated mice.

**Figure S7 Mitotic index and apoptosis in L0627 tumors**


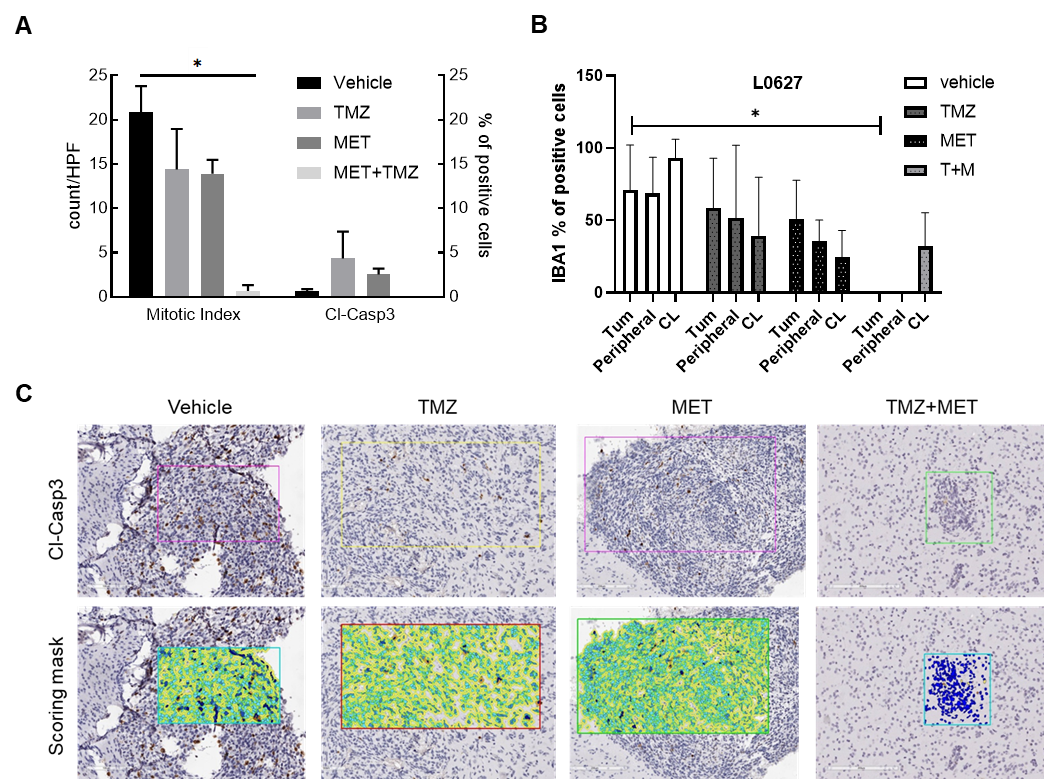


**Supplementary Figure 7.** Glioma cell viability was investigated in explanted brain counting mitotic cells in two high power fields per sample and performing a staining with an anti-cleaved Caspase 3 (Cl-Casp3) antibody. In addition, IBA1 expression was evaluated. **A)** The number of mitotic cells or the percentage of cleaved-Caspase 3 positive cells was scored (n=4 for vehicle, n=5 for TMZ, n=5 for MET and n=2 for TMZ+MET). One-way ANOVA analysis followed by Tukey’s multiple comparison test was performed; * p = 0.0142. Bars, mean±SEM. **B)** Quantification of IBA1 marker expressed as percentage of positive cells in the inner part of the tumor (Tum), in tumor-brain border (Peripheral) and in the brain region contralateral to the tumor (CL). Two-way ANOVA analysis followed by Bonferroni’s multiple comparison test; * p = 0.0414. **C)** Representative images of glioma stained for cleaved-Caspase 3 presence; bottom, scoring mask of the digital algorithm.

**Figure S8 [^18^F]VC701 uptake in contralateral area to tumor compared to brain uptake of healthy animals**


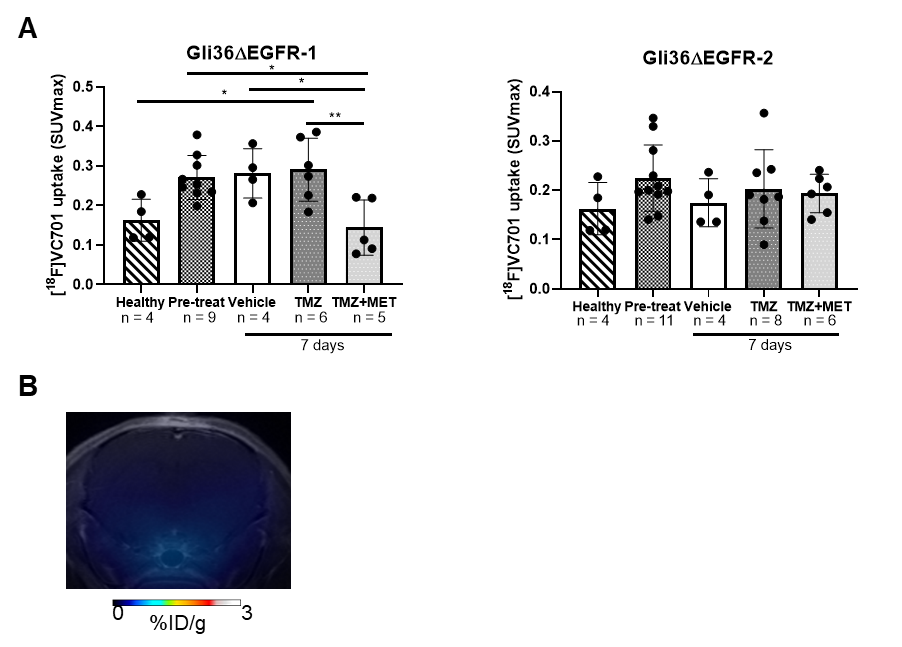


**Supplementary Figure 8.** [^18^F]VC701 uptake in contralateral area to tumor in healthy mice and in GBM mouse models. **A**, after 7 days of TMZ+MET treatment, [^18^F]VC701 uptake in contralateral area to tumor in Gli36ΔEGFR-1 tumor-bearing mice was significantly decreased compared to pre-treatment condition and to vehicle and TMZ-treated mice and became similar to that of healthy mice. One-way Anova analysis followed by Tukey’s multiple comparisons test was performed. * p < 0.05, ** p < 0.01. In Gli36ΔEGFR-2 tumor-bearing mice no difference was detected. Uptake data are expressed as SUVmax. **B**, Representative [^18^F]VC701 PET image of a brain of a healthy mouse. Color scale is expressed as %ID/g.

**Figure S9 [^18^F]VC701 uptake in L0627 tumor and in contralateral area compared to brain uptake of healthy animals**


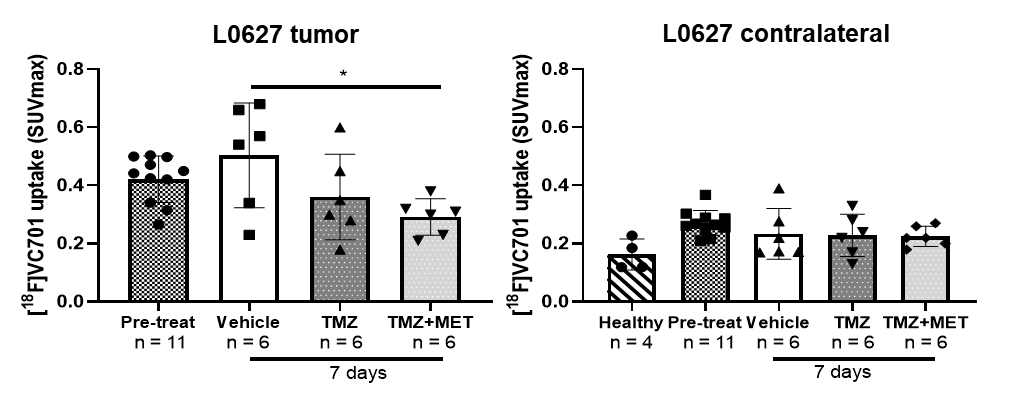


**Supplementary Figure 9** [^18^F]VC701 uptake in L0627 tumors and in the contralateral area to tumor in healthy mice and in GBM mouse models. After 7 days of TMZ+MET treatment, [^18^F]VC701 uptake in tumors was significantly decreased compared to vehicle-treated mice. * p < 0.05. One-way Anova analysis followed by Tukey’s multiple comparisons test was performed. In contralateral area to the tumor, we didn’t observe any change. Uptake data are expressed as SUVmax.

**Supplementary table 1**

Summary table of Gli36ΔEGFR-1 tumor bearing mice

| **Mouse id** | **Treatment** | **Survival** | **Survival from cell injection (days)** |
| --- | --- | --- | --- |
| M1 | Vehicle | Y | 14 |
| M6 | Vehicle | Y | 18 |
| M9 | Vehicle | Y | 14 |
| M25 | Vehicle | Y | 14 |
| M30 | Vehicle | Y | 17 |
| M3G | Vehicle | Y | 18 |
| M5G | Vehicle | Y | 18 |
| M12G | Vehicle | Y | 18 |
| M16G | Vehicle | Y | 17 |
| M9L | Vehicle | N | 16 |
| M11L | Vehicle | N | 16 |
| M12L | Vehicle | N | 16 |
| M2 | TMZ | Y | 43 |
| M7 | TMZ | Y | 60 |
| M12 | TMZ | Y | 90 |
| M19 | TMZ | Y | 90 |
| M24 | TMZ | Y | 63 |
| M1G | TMZ | N | 36 |
| M4G | TMZ | N | 36 |
| M6G | TMZ | N | 36 |
| M7G | TMZ | N | 36 |
| M1L | TMZ | N | 16 |
| M2L | TMZ | N | 16 |
| M3L | TMZ | N | 16 |
| M4L | TMZ | N | 16 |
| M3 | MET | Y | 14 |
| M8 | MET | Y | 15 |
| M13 | MET | Y | 17 |
| M18 | MET | Y | 13 |
| M23 | MET | Y | 14 |
| M27 | MET | Y | 15 |
| M4 | TMZ + MET | Y | 90 |
| M10 | TMZ + MET | Y | 90 |
| M14 | TMZ + MET | Y | 90 |
| M22 | TMZ + MET | Y | 90 |
| M29 | TMZ + MET | Y | 90 |
| M2G | TMZ + MET | N | 36 |
| M8G | TMZ + MET | N | 36 |
| M9G | TMZ + MET | N | 36 |
| M11G | TMZ + MET | N | 36 |
| M5L | TMZ + MET | N | 16 |
| M6L | TMZ + MET | N | 10 * |
| M7L | TMZ + MET | N | 10 * |
| M8L | TMZ + MET | N | 16 |

Y = Yes; N =No. When the sacrifice has been programmed after a cycle of therapy (36 d) or one week after the beginning of therapy (16 d), mice have not been used for survival curve. * mice sacrificed earlier for an issue in the administration of the drugs.

**Supplementary table 2**

Summary table of Gli36ΔEGFR-2 tumor bearing mice

| **Mouse id** | **Treatment** | **Survival** | **Survival from cell injection (days)** |
| --- | --- | --- | --- |
| M1 | Vehicle | Y | 14 |
| M6 | Vehicle | Y | 18 |
| M11 | Vehicle | Y | 17 |
| M25 | Vehicle | Y | 13 |
| M30 | Vehicle | Y | 14 |
| M2G | Vehicle | Y | 17 |
| M5G | Vehicle | Y | 14 |
| M12G | Vehicle | Y | 17 |
| M15G | Vehicle | Y | 17 |
| M9L | Vehicle | N | 14 |
| M5L | Vehicle | N | 16 |
| M11L | Vehicle | N | 14 |
| M12L | Vehicle | N | 14 |
| M2 | TMZ | Y | 21 |
| M7 | TMZ | Y | 21 |
| M12 | TMZ | Y | 21 |
| M19 | TMZ | Y | 20 |
| M24 | TMZ | Y | 16 |
| M29 | TMZ | Y | 20 |
| M3G | TMZ | Y | 18 |
| M4G | TMZ | Y | 18 |
| M6G | TMZ | Y | 18 |
| M7G | TMZ | Y | 18 |
| M10G | TMZ | Y | 16 |
| M16G | TMZ | Y | 18 |
| M18G | TMZ | Y | 18 |
| M1L | TMZ | N | 16 |
| M2L | TMZ | N | 16 |
| M4L | TMZ | N | 15 |
| M7L | TMZ | N | 16 |
| M3 | MET | Y | 18 |
| M13 | MET | Y | 15 |
| M18 | MET | Y | 13 |
| M23 | MET | Y | 14 |
| M28 | MET | Y | 13 |
| M4 | TMZ + MET | Y | 15 |
| M8 | TMZ + MET | Y | 24 |
| M14 | TMZ + MET | Y | 22 |
| M17 | TMZ + MET | Y | 13 |
| M22 | TMZ + MET | Y | 27 |
| M27 | TMZ + MET | Y | 24 |
| M1G | TMZ + MET | N | 10 * |
| M8G | TMZ + MET | N | 10 * |
| M9G | TMZ + MET | N | 10 * |
| M11G | TMZ + MET | N | 18 |
| M13G | TMZ + MET | N | 16 |
| M14G | TMZ + MET | N | 18 |
| M10L | TMZ + MET | N | 16 |
| M6L | TMZ + MET | N | 16 |
| M3L | TMZ + MET | N | 14 |
| M8L | TMZ + MET | N | 14 |

Y = Yes; N =No. When the sacrifice has been programmed after one week from the beginning of therapy (14-16 d), mice have been not used for survival. * mice sacrificed before for a problem in the administration of the drugs.
